# Heavy Metals in Dialysis Fluid and Blood Samples from Hemodialysis Patients in Dialysis Centers in Baghdad, Iraq

**DOI:** 10.5696/2156-9614-10.27.200901

**Published:** 2019-08-19

**Authors:** Yasamen Raad Humudat, Saadi Kadhim Al-Naseri

**Affiliations:** Environment and Water Directorate, Ministry of Science and Technology, Baghdad, Iraq

**Keywords:** chronic kidney disease, dialysis fluid, hemodialysis, heavy metals

## Abstract

**Background.:**

The kidney is the first target organ of heavy metal toxicity due to its capacity to reabsorb and accumulate divalent metals. Hemodialysis therapy is used to purify the blood of individuals with impaired kidney function.

**Objective.:**

The aim of the present study was to evaluate the relationship between dialysis fluid quality and the health of hemodialysis patients.

**Methods.:**

A field sampling program was conducted to collect blood samples from 320 hemodialysis patients (56% males and 44% females) in order to examine the concentrations of heavy metals that typically occur in municipal water in Baghdad (aluminum (Al), copper (Cu), lead (Pb), and zinc (Zn)), and explore associations with the same metals in dialysis fluid collected from four major dialysis centers in Baghdad hospitals for a period of one year (2018).

**Results.:**

The results showed that the dialysis fluid quality was not in compliance with international standards. The dialysis fluid in 63% of the samples contained high Al concentrations, while Cu and Zn concentrations were within international standards. Lead concentrations were elevated in dialysis fluid in some hospitals as well.

**Discussion.:**

The average blood levels of biologically important heavy elements were significantly varied in hemodialysis patients when compared with local reference values.

**Conclusions.:**

Since both deficiency and excess elements are potentially harmful, the hypothesis that heavy element status affects the risk of adverse clinical outcomes is a worthy investigation.

**Participant Consent.:**

Obtained

**Ethics Approval.:**

The study was approved by the Baghdad Ethics Committee of the Iraqi Ministry of Health and Environment.

**Competing Interests.:**

The authors declare no competing financial interests.

## Introduction

Chronic kidney disease is a form of kidney disease in which kidney function is gradually lost over a period of time, and has a major impact on health-related quality of life and medical service use.^1^–^3^ Exposure to heavy metals is associated with chronic kidney disease.^4^ The kidney is the first target organ of heavy metal toxicity due to its capacity to reabsorb and accumulate divalent metals.^5^

Aluminum (Al) can be toxic to those with chronic renal failure due to modified Al metabolism and present several health risks, including dementia.^6^ Lead (Pb) exposure has well known health effects, including tubulointerstitial nephropathy that can lead to kidney failure.^7^ Copper deficiency can cause anemia and neutropenia.^8^ Zinc deficiency causes or contributes to a number of infections and non-specific conditions commonly observed in patients with hemodialysis, including anorexia, dysgeusia, and impeded cognitive function.^9^

Aluminum compounds are the conventional coagulant used in the treatment of the municipal water, while the other metals (Pb, Cu and Zn) can be found in municipal water due to their existence in most water pipes and fittings used in Baghdad.^10^ This raises questions of possible adverse effects on the health of hemodialysis patients.

During hemodialysis, essential functions of the kidney, such as elimination of water and metabolic waste, as well as electrolyte and acid/base correction, are replaced by an artificial purification system or a dialyzer.^11^ Toxic elements must be maintained within a limited physiological range.^12^

Dialysis fluid or dialysate is used in dialysis to adjust the composition of extracellular fluid and maintain homeostasis of the body. Dialysate is produced by mixing clean reverse osmosis water with acid and base concentrate.^13^

During treatment, hemodialysis patients are typically exposed to an extremely large amount of water, more than 90 to 192 L of water per session, two or three times a week.^14^ Therefore, the quality of the water utilized for dialysis fluid preparation is very important to prevent diffusion of contaminants into the patient's bloodstream.^15^ Because dialysis uses a large amount of water, even a low concentration of contaminants can pose health risks. Some substances may cause conditions such as anemia or pyrogenic responses, and some may accumulate to toxic concentrations, resulting in long-term physical damage, while other substances are immediately toxic and may cause death.^16^

Epidemiological studies have shown a strong correlation between exposure to toxic elements and chronic kidney injury.^17^ However, no previous studies have examined these elements in the water used for hemodialysis treatment. A study conducted in Poland showed that dialysis fluids seem to be safe for hemodialysis patients but emphasized the importance of controlling element levels in dialysis fluid to prevent complications.^18^

At present, there is no national dialysis fluid quality standard in Iraq. Thus, the standards set by the ISO 23500-2:2019 were adopted to compare the results of the present study evaluating the quality of dialysis water used for hemodialysis treatment and the relationship to hemodialysis patient health.^19^

## Methods

The present study was carried out in 2018, with voluntary participation of 320 hemodialysis patients identified as having chronic kidney disease without hepatitis virus infection. Viral hepatitis patients were excluded because they were treated in isolation units.

**Abbreviations**
ISO: International Organization for Standardization
LSD: Least significant difference

Study participants included male and female subjects registered for regular hemodialysis treatment in four hemodialysis centers in Baghdad, Iraq: Al-Kindi, Baghdad Teaching, Kadhimiya (also now known as Al-Imammain Al-Kadhimain), and Al-Yarmouk. The study took place during one year across the four seasons: Spring (March 1, 2018 to May 31, 2018), Summer (June 1, 2018 to August 31, 2018), Autumn (September 1, 2018 to November 30, 2018), and Winter (December 1, 2018 to February 28, 2019), including 20 patients from each hospital during each of these four seasons.

### Ethics approval

The study was approved by the Baghdad Ethics Committee of the Iraqi Ministry of Health and Environment. All participants in the study gave their written permission to access their medical records, blood sampling, and anonymous use of their samples.

### Demographic information

Participant demographic information was collected using a researcher-made questionnaire. Information was further verified by cross checking with medical records.

### Blood sampling

Blood samples were collected once from each patient during the hemodialysis session. The samples were collected from the venous port of the hemodialysis catheter before adding heparin to the whole blood samples of regular hemodialysis patients. The first 5 IU/ml of blood were discarded to avoid activation of coagulation due to puncture trauma.^20^ Serum was obtained by centrifugation at 3000 rpm for 10 minutes and stored at −20°C until use.

### Water sampling

Water samples were collected once from the same four dialysis centers from which blood samples were taken at the same time. Water samples (250 ml) were collected and transported to the laboratory for chemical analysis in a cool box.

### Heavy metals analysis

The analysis of heavy metals was done using the graphite furnace atomic absorption spectrometry method (AA-7000, Shimadzu, Japan) in the laboratories of the Iraqi Ministry of Science and Technology.

### Statistical analysis

Statistical analysis was carried out using analysis of variance and least significant difference (LSD) methods to compare different factors in study parameters at a p-value of 0.05. Microsoft Excel 2010 was used to calculate the results. The statistical correlation coefficient was calculated between the heavy metal concentrations across all blood samples with their concentration in the dialysis fluid. The calculations took into consideration patient gender (male/female) and calculations were performed separately for each gender. Data were expressed as mean±SD and percentage (%).^21^

## Results

Table 1 presents the demographics of the 320 studied patients. Approximately 56% of patients were male and 44% were female, and there were significant differences between males and females in age and weight (male patients were older and heavier). Body temperature showed no significant differences between males and females, and only 4% of the male patients were smokers. The education level of both males and females was relatively low.

**Table 1:** Patient Demographics

**Variables and category**	**Males**	**Females**	**Difference at p <5%**
No. of patients ±SD (%)	178± 5.8 (56%)	142±5.8 (44%)	-
Age range (yrs) (mean±SD)	19–76 (53±13)	16–85 (49 ±16)	(S)
Weight range (kg) (mean±SD)	37–135 (76±16.9)	35–120 (66 ±16.6)	(S)
Temperature range (°C) (mean±SD)	36–38 (37 ±0.6)	36–38 (37 ±0.6)	(NS)
Current smoking % (number±SD)	4% (12 ±0.9)	0% (0±0)	-
Education level % (high school or higher) (number)	10% (32)	8% (26)	(NS)

Abbreviations: NS, not significant; S, significant

### Heavy metals in dialysis fluid

Table 2 shows the chemical analysis of water samples over the four seasons of monitoring for the four dialysis centers. The results were compared to standards published by the ISO, and results in Table 2 with values higher than the ISO standard are expressed in bold.

**Table 2:** Seasonal Variation for Heavy Metals from Dialysis Fluid Samples from Hospital Dialysis Centers by Season

**Al level (mg/l)** **ISO level = 0.01 mg/l**	**Spring**	**Summer**	**Autumn**	**Winter**	**LSD value**

Al-Kindi	**0.05**	**0.071**	0.004	0.004	0.044^*^
Baghdad Teaching	**0.03**	0.004	0.004	**0.033**	NS
Al-Imamain Al-Kadhimain	**0.045**	0.004	**0.05**	**0.03**	NS
Al-Yarmouk	BDL	**0.013**	**0.03**	**0.02**	NS
LSD value	NS	NS	NS	NS	—

**Cu level (mg/l)** **ISO level = 0.1 mg/l**	**Spring**	**Summer**	**Autumn**	**Winter**	**LSD value**

Al-Kindi	0.001	0.001	0.001	0.002	NS
Baghdad Teaching	0.001	0.003	0.003	0.003	NS
Al-Imamain Al-Kadhimain	0.05	0.003	0.001	0.001	NS
Al-Yarmouk	0.04	0.001	0.001	0.001	NS
LSD value	NS	NS	NS	NS	—

**Pb level (mg/l)** **ISO level = 0.005 mg/l**	**Spring**	**Summer**	**Autumn**	**Winter**	**LSD value**

Al-Kindi	**0.006**	0.001	0.001	0.001	NS
Baghdad Teaching	0.002	0.0001	0.001	0.001	NS
Al-Imamain Al-Kadhimain	**0.12**	0.001	0.001	0.001	0.068^*^
Al-Yarmouk	0.0002	0.002	0.001	0.001	NS
LSD value	0.066^*^	NS	NS	NS	—

**Zn level (mg/l)** **ISO level = 0.1 mg/l**	**Spring**	**Summer**	**Autumn**	**Winter**	**LSD value**

AL-Kindi	0.0002	BDL	BDL	0.007	NS
Baghdad Teaching	0.002	0.04	BDL	0.0013	NS
Al-Imamain Al-Kadhimain	0.0001	0.0012	0.0012	0.0012	NS
Al-Yarmouk	0.02	0.001	0.0012	0.0012	NS
LSD value	NS	NS	NS	NS	—

^*^ Significant (p <5%)

Abbreviations: NS, not significant; BDL, below detection limit, LSD, least significant difference Bolded figures are higher than ISO standard values^19^

The results showed that 63% of dialysis fluid samples had aluminum (Al) levels exceeding the international standard guideline, while all copper (Cu) and zinc (Zn) concentrations were within the international standard at an acceptable level. Approximately 6% of the samples showed lead (Pb) concentrations higher than the international standard.

The statistical analysis showed no significant differences in Al, Cu or Zn concentrations across the four hospitals, with the exception of Pb concentrations in the spring, where LSD=0.068 (p <5%). In addition, seasonal variation showed no significant differences with the exception of Al in Al-Kindi, and Pb in Al-Imamain Al-Kadhimain, with LSD values of 0.044 and 0.068, respectively.

### Heavy metals in blood samples

Table 3 summarizes the results of the mean heavy metal concentrations (mg/l) reported as mean±SD in blood samples, together with their seasonal and spatial variation. Bold values in Table 3 indicate levels exceeding normal values.^22^

**Table 3:** Heavy Metal Concentrations (mg/l) in Hemodialysis Patient Blood Samples Across Hospital Dialysis Centers by Season

**Al level (mg/l)** **normal value <0.02^22^**	**Spring**	**Summer**	**Autumn**	**Winter**	**LSD value**

Al-Kindi	0.10±0.06	0.01±0.01	0.14±0.11	0.21±0.16	0.166^*^
Baghdad Teaching	0.15±0.14	0.05±0.03	0.23±0.19	0.09±0.12	0.149^*^
Al-Imamain Al-Kadhimain	0.11±0.07	0.025±0.01	0.07±0.02	0.11±0.08	NS
Al-Yarmouk	0.14±0.14	0.33±0.2	0.16±0.09	0.22±0.23	NS
LSD value	NS	0.109^*^	0.177^*^	NS	—

**Cu level (mg/l)** **normal value 0.8–1.5^22^**	**Spring**	**Summer**	**Autumn**	**Winter**	**LSD value**

Al-Kindi	0.15±0.03	0.45±0.21	0.17±0.13	0.34±0.49	0.107^*^
Baghdad Teaching	0.24±0.15	0.17±0.06	0.14±0.05	0.24±0.06	NS
Al-Imamain Al-Kadhimain	0.30±0.13	0.24±0.12	0.43±0.11	0.18±0.04	0.204^*^
Al-Yarmouk	0.22±0.07	0.35±0.08	0.16±0.03	0.42±0.49	0.127^*^
LSD value	0.119^*^	0.184^*^	0.116^*^	0.109^*^	—

**Pb level (mg/l)** **normal value <0.05^22^**	**Spring**	**Summer**	**Autumn**	**Winter**	**LSD value**

Al-Kindi	0.01±0.01	0.01±0.004	0.02±0.02	0.01±0.01	NS
Baghdad Teaching	0.03±0.03	0.01±0.01	0.02±0.01	0.01±0.01	NS
Al-Imamain Al-Kadhimain	0.02±0.04	0.001±0.00	0.01±0.01	0.01±0.01	NS
Al-Yarmouk	0.01±0.01	0.02±0.01	0.01±0.01	0.01±0.01	NS
LSD value	NS	NS	NS	NS	—

**Zn level (mg/l)** **normal value 0.7–1.2^22^**	**Spring**	**Summer**	**Autumn**	**Winter**	**LSD value**

AL-Kindi	1.5±0.16	0.84±0.14	0.67±0.24	0.81±0.58	0.461^*^
Baghdad Teaching	0.75±0.47	1.27±0.53	0.65±0.54	0.86±0.19	0.458^*^
Al-Imamain Al-Kadhimain	1.03±0.33	0.75±0.28	0.71±0.23	0.65±0.21	0.333^*^
Al-Yarmouk	1.5±0.16	0.84±0.14	0.66±0.24	1.01±0.6	0.472^*^
LSD value	0.502^*^	0.471^*^	NS	0.328^*^	—

^*^ Significant (P<5%)

Abbreviation: NS, not significant, LSD, least significant difference

The results showed that 81% of the blood samples had elevated Al concentrations (0.13±0.08 mg/l) when compared to normal values. The statistical analysis showed significant differences in Al levels among the hospitals in the summer and autumn (LSD=0.109 and 0.177, respectively). Furthermore, seasonal significant differences in mean blood Al levels were detected in Al-Kindi and Baghdad Teaching hospital at p <5%, with LSD of 0.166 and 0.149, respectively.

The levels of Cu (0.26±0.10 mg/l) in blood samples were lower than normal values. The statistical analysis showed significant differences in mean Cu levels across the studied hospitals during the study period, and there were significant seasonal differences in all the studied hospitals, except in Baghdad Teaching hospital, at p <5%.

All Pb concentration results (0.013±0.01 mg/l) were within normal levels compared with local normal values, and there were no significant differences across seasons and hospitals at p <5%.

Finally, Zn levels were observed to fluctuate, as 18% of the tested samples were higher than the normal range, and 25% of the samples were lower than the normal range. Statistical analysis showed significant differences in mean Zn levels across hospitals except in the autumn period, and there were significant seasonal differences among all the hemodialysis centers studied at p <5%.

### Correlation of heavy metals between dialysis fluid and blood samples

Table 4 presents the relationship between heavy metal concentrations in hemodialysis patients' blood samples at the time of dialysotherapy and heavy metal concentrations in water samples from dialysis fluid. The correlation coefficient took into consideration the effects on males and females separately. The correlation coefficients for Al for both males and females were negative and insignificant (p-values >0.05). The correlation coefficients for Zn and Cu for both males and females were weak and insignificant (p-values >0.05). Lead showed a strong and significant correlation (p-value <0.001) for males with a correlation coefficient of 0.83, while there was an insignificant correlation for females.

**Table 4:** Correlation Coefficient Between Metal Concentrations in Dialysis Fluid and Patient Blood Samples

**Metals**	**Correlation coefficient-r**		**p-value**	

	**Males**	**Females**	**Males**	**Females**
Al	−0.15	−0.3	0.58	0.22
Cu	0.17	0.19	0.51	0.48
Pb	0.83	−0.06	0.00005^*^	0.82
Zn	0.28	0.32	0.28	0.19

^*^Significant.

## Discussion

The results of the present study showed that the concentrations of each of the heavy metals in the two types of samples (dialysis fluid and blood) had different trends. There have been no previous studies assessing the relationship between dialysis water quality and the health of hemodialysis patients in the study area.

According to Table 1, some of the patients were characterized by low body weight, which could be due to hospitalization for hemodialysis which can have a negative nutritional impact.^23^ There were small differences in the body temperature of hemodialysis patients, but these differences were not significant. However, low body temperatures in patients with hemodialysis are not unusual.^24^

Finally, the results showed that the majority of patients in the present study had low levels of formal education.

Patients on chronic hemodialysis are at high risk of developing heavy metal imbalances.^25^ All of the hemodialysis centers in the present study showed very high Al content in the dialysis fluid, while some showed an elevated concentration of Pb. Aluminum concentrations in the majority of the blood samples were higher than the normal values. However, the results in Table 4 showed an insignificant and negative correlation coefficient for Al for both males and females. This indicates that elevated blood concentrations of Al came from sources other than dialysis fluid. Elevated aluminum concentrations in this population are expected, due to the current practice of using Al sulfate as a coagulant for clarifying municipal water (the feed water to the dialysis centers). This finding disagrees with the previously mentioned Polish study, where the averaged Al concentration did not exceed the acceptable maximum level for water of 0.01 mg/l and suggested that Al would not be retained in a patient's body.^18^

The majority of the dialysis fluid samples were within the acceptable level for Pb concentration at Baghdad hospitals, which contributed to normal Pb concentration values in hemodialysis patients' blood samples. However, in the Spring (March to May) two hospitals showed elevated Pb concentrations and blood samples from male patients from these hospitals were elevated as well. Table 4 demonstrates a significant correlation coefficient for Pb concentration with blood samples of male patients. The reasons behind the Pb elevated concentration remains unclear and requires further investigation. The Pb concentration results in the present study agree well with other studies which found that the blood Pb level increases in hemodialysis patients when it exists at an elevated concentration in dialysis fluid.^26^

The prevalence of Cu in the dialysis fluid was in compliance with the international standards, and there was an insignificant correlation with the Cu concentration in blood samples. This result disagrees with previous studies of water samples conducted by the same author, which found that Cu levels exceeded international standards.^10^ Some studies showed that blood Cu levels were within a normal range or lower^27^, while in other studies Cu levels were higher.^8^,^28^

Zinc concentrations were in compliance with the international standard during the entire study period and in all the hospitals in the present study. The results for Zn levels disagree with a previous study by Al-Naseri *et al.* which showed non-compliance with international standards for the same hospitals.^10^ The results were in agreement with the results of a previous study which showed that 58% of hemodialysis patients had Zn deficiency (Zn level of less than 0.7 mg/l).^26^,^29^

### Limitations

There is a need to study pre- and post-dialysis serum biochemical measurements, and the relationship to morbidity risk indicators in hemodialysis patients.

## Conclusions

The average blood levels of biologically important heavy elements were significantly varied in hemodialysis patients when compared with local reference values. Since both deficiency and excess elements are potentially harmful, the hypothesis that heavy element status affects the risk of adverse clinical outcomes is a worthy investigation. There were insignificant correlations between Cu, Zn, and Al in dialysis fluid and blood samples, while Pb concentration showed a significant correlation with blood samples collected from males only.
